# Longitudinal Follow-Up of the Psychological Well-Being of Patients with Colorectal Cancer: Final Analysis of PICO-SM

**DOI:** 10.3390/curroncol31120582

**Published:** 2024-12-11

**Authors:** Konstantinos Kamposioras, Panagiotis Ntellas, Katerina Dadouli, Eleftherios Christodoulis, Marios Adamou, Daniel Anderson, Anup Shanthappa, Jacqueline Connell, Joseph Williams, Lilly Simpson, Theodora Germetaki, Michael Braun, Jorge Barriuso, Jurjees Hasan, Saifee Mullamitha, Kalena Marti, Mark Saunders, Kok Haw Jonathan Lim

**Affiliations:** 1 Department of Medical Oncology, The Christie NHS Foundation Trust, Manchester M20 4BX, UK; 2 Department of Medical Oncology, University Hospital of Ioannina, 45500 Ioannina, Greece; 3 Laboratory of Hygiene and Epidemiology, Faculty of Medicine, University of Thessaly, 41222 Larissa, Greece; 4 School of Human and Health Sciences, Queensgate, University of Huddersfield, Huddersfield HD1 3DH, UK; 5 Department of Psycho-Oncology, The Christie NHS Foundation Trust, Manchester M20 4BX, UK; 6 Division of Pharmacy and Optometry, School of Health Sciences, The University of Manchester, Manchester M13 9PT, UK; 7 Division of Cancer Sciences, Faculty of Biology, Medicine and Health, The University of Manchester, Manchester M20 4BX, UK; 8 Manchester Cancer Research Centre, Manchester M20 0GJ, UK; 9 Department of Clinical Oncology, The Christie NHS Foundation Trust, Manchester M20 4BX, UK; 10 Advanced Immunotherapy and Cell Therapy Team, Department of Medical Oncology, The Christie NHS Foundation Trust, Manchester M20 4BX, UK

**Keywords:** anxiety, depression, psychological distress, colorectal cancer, COVID-19

## Abstract

PICO-SM was a prospective longitudinal study investigating the psychological impact of the COVID-19 pandemic on patients with colorectal cancer treated in a large UK tertiary cancer centre. Here, we present the impact of the third wave of the pandemic (December 2021 to February 2022), when the Omicron variant became prevalent in the UK, and the complete longitudinal comparison across the entire duration of this study. Patients were invited to complete a questionnaire, including screening psychometric tools. In total, n = 312 patients were included in the final analysis. Specifically, in this Omicron-predominant wave, n = 96 patients were studied in detail: the mean age was 64 years, 64% were male, 33% reported poor well-being, 27% anxiety, 11% depressive symptoms, and 3% trauma-related symptoms. The participants who had investigations cancelled (OR 9.22, 95% CI 1.09–77.85; *p* = 0.041) or felt that the pandemic would affect their mental health (OR 3.82, 95% CI 1.96–7.44; *p* < 0.001) had an increased risk of anxiety according to a multivariate analysis. Similarly, independent predictors of poor well-being included concern that the pandemic would affect their cancer treatment (OR 4.59, 95% CI 1.03–20.56; *p* = 0.046) or mental health (OR 3.90, 95% CI 1.38–11.03; *p* = 0.010). The psychological distress experienced by patients, particularly anxiety, remained high during the third wave of the COVID-19 pandemic. These results align with our previously reported findings, emphasising the importance of continuing cancer treatment amidst an ongoing humanitarian emergency.

## 1. Introduction

The emergence of the coronavirus disease 2019 (COVID-19) pandemic dramatically impacted every aspect of cancer care, including the diagnosis of new patients with cancer, treatment decisions, and general access to treatment [1,2]. Specifically, in the management of colorectal cancer in the United Kingdom (UK), there were significant disruptions in the screening and diagnostic pathways, resulting in a reduction in the number of surveillance endoscopies, the cancellation of elective surgeries, and changes in the surveillance schedule and radiotherapy and/or systemic treatment regimens [3,4,5].

Previous reports have highlighted that patients with colorectal cancer experience clinically significant levels of anxiety and depression [6,7]. In a meta-analysis of studies conducted between 2005 and 2020, the estimated prevalence of anxiety was 18.9% (95% CI 15.2–22.7%) and that of depression was 22.9% (95% CI 13.8–23.0%) [6]. In the most recent years preceding to the COVID-19 pandemic, the prevalence of anxiety and depression was 25.9% and 18.2%, respectively [6]. It is believed that these psychological distress levels would have been exacerbated further by the pandemic acting as an additional stressor [8,9].

At the beginning of the COVID-19 pandemic, we and others contemporaneously reported that adaptations in cancer care had resulted in emotional distress, with at least one in five patients at risk of anxiety [3,10]. This was lower than initially hypothesised, with most patients reporting that continuing with their respective cancer treatment remained a top priority [11]. Therefore, in response to the predicted long-term repercussions of the COVID-19 pandemic on cancer care, we established the ‘Psychological Impact of COVID-19 on Patients with Solid Malignancies: A Single Institution Survey Study’ (PICO-SM) at The Christie NHS Foundation Trust (Manchester, UK) [11]. This was a prospective longitudinal observational study on the mental health and well-being of patients with colorectal cancer who were newly diagnosed and/or received their cancer care during this unprecedented era (2021–2022) [11]. In the first cohort recruited to PICO-SM in 2021, we observed that patients with pre-existing mental health conditions and those with the perception that the pandemic would affect their cancer management were at higher risk of experiencing anxiety and depressive symptoms and have poor well-being [11].

Here, we present the results from the second cohort of PICO-SM, focusing on psychological effects during the third wave of the pandemic in 2022, in the era of “living with COVID-19” with no national restrictions, marked by the prevalence of the Omicron variant, increasing the risk of hospitalisations with COVID-19 infection [12].

## 2. Materials and Methods

### 2.1. Participants

PICO-SM was a single-centre prospective study that included patients with colorectal cancer attending the specialised lower gastrointestinal cancer clinics at The Christie NHS Foundation Trust, carried out via convenience sampling. The Christie is a large publicly funded comprehensive cancer centre located in the Northwest of England (Manchester, UK). For Cohort 1, patients were invited to participate in a longitudinal survey between April 2021 and January 2022, as previously reported [11]. For Cohort 2, a different group of patients were identified through a review of the list of clinics, recruited in person or remotely (via telephone or video consultations) between 6 December 2021 and 21 February 2022 (time point 1). Participants who agreed to be contacted again at a later date were invited to participate 6 months after completing the first survey, between 21 June 2022 and 13 October 2022 (time point 2). All eligible participants were ≥18 years and able to fully comprehend the patient information sheet. Participation was entirely voluntary, and no financial incentive was offered for completion of the survey.

### 2.2. Study Design and Objectives

This observational survey study, as previously described [11], consisted of a 30-item questionnaire used to capture basic demographics, the perceived impact of COVID-19 infection on mental health, coping strategies, and the support received.

The primary objective for Cohort 2 of PICO-SM was to evaluate the levels of anxiety, depressive symptoms, trauma-related symptoms, and general well-being amongst patients with colorectal cancer using the following validated self-reported screening tools: Generalized Anxiety Disorder scale (GAD-7) for anxiety [13], Patient Health Questionnaire-9 (PHQ-9) for depression [14], Primary Care Post-Traumatic Stress Disorder-5 (PC-PTSD-5) for probable PTSD [15], and World Health Organization Well-being Index (WHO-5) for mental well-being [16]. The secondary objective of the study was to understand how the mental health burden, as reflected by these measures, evolved throughout the course of the COVID-19 pandemic and further interrogate any clinically meaningful differences between the two cohorts of the study.

### 2.3. Statistical Analysis

Descriptive analyses for all the variables were carried out using R language (R Core Team: R: A Language and Environment for Statistical Computing Vienna, Austria: Foundation for Statistical Computing; available from: https://www.r-project.org/) and data represented using GraphPad Prism version 9.3.0 for Mac (San Diego, CA, USA). Data were expressed as mean ± standard deviation (continuous variables), or as frequencies (n) and percentages (%) (categorical variables). A Chi-square test or Fisher’s exact test and Student’s *t*-Test or Mann–Whitney U Test were used to analyse categorical and continuous variables, respectively. McNemar’s test or related-samples Wilcoxon signed-rank test was used to compare the participants’ answers between time points 1 and 2 of the longitudinal survey study.

A multivariate analysis was conducted using logistic regression for binary outcomes (anxiety (GAD-7 score ≥ 5), depressive symptoms (PHQ-9 score ≥ 10), and poor well-being (WHO-5 < 50)), and odds ratios (OR) with 95% confidence intervals (CIs) were calculated. Variables with *p* < 0.10 on univariate analyses were included in the final model. A multivariable analysis was not conducted for PTSD-related symptoms due to the low incidence of such events. Pearson’s bivariate correlation analysis was used to validate the association between the following key outcome measures: GAD-7, PHQ-9, PC-PTSD-5, and WHO-5. For all the analyses, a 5% significance level was set, and the *p* values were two-tailed.

## 3. Results

### 3.1. Survey Participants

A total of n = 247 patients were invited to participate in Cohort 2 of PICO-SM, and n = 96 patients consented to be included in the study; the response rate was 38.9%. The majority of survey participants was male (n = 61/96, 63.6%), with a mean age of 64 years [Table 1]. Most were White British (n = 89/96, 92.7%), representative of the ethnic distribution of the local population (Census 2021, Office for National Statistics) [Table 1]. More than half of the participants (n = 64/96, 66.7%) reported a previous diagnosis of a mental health condition prior to the onset of the pandemic, most commonly anxiety and/or depression [Table 1]. The presence of a condition that may increase the personal risk of contracting COVID-19, such as diabetes and respiratory or cardiovascular comorbidities, was reported by 31.3% (n = 30/96) of the participants [Table 1]. The majority of participants had undergone testing for COVID-19, out of which n = 13/91 (14.3%) had tested positive for COVID-19 infection at some point, with n = 3 participants requiring hospitalisation for COVID-19. Eighty-seven patients (90.6%) were invited to participate in the longitudinal follow-up survey 6 months later (time point 2); nine patients died during the follow-up period of the study.

### 3.2. The Mental Health Status of Cohort 2 of PICO-SM

**Figure 1:**
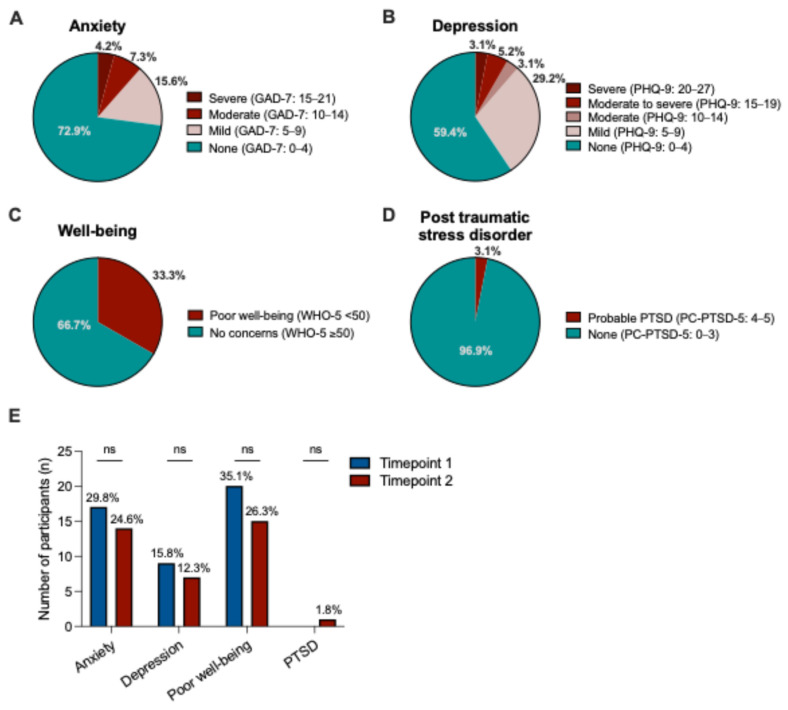
The psychological impact of the COVID-19 pandemic on the prevalence of (**A**) anxiety, (**B**) depressive symptoms, (**C**) poor well-being, and (**D**) trauma-related symptoms in patients with colorectal cancer (n = 96). (**E**) Comparison of the key outcome variables for the longitudinal subgroup of n = 57 participants followed-up across two time points. Abbreviations: GAD-7, Generalized Anxiety Disorder scale; PC-PTSD-5, Primary Care Post-Traumatic Stress Disorder-5; PHQ-9, Patient Health Questionnaire-9; WHO-5, World Health Organization Well-being Index; and ns, not significant.

Among the participants who completed the psychometric screening questionnaire, 33.3% (n = 32/96) reported poor well-being (WHO-5 < 50), 27.1% (n = 25/96) were at risk for mild to severe anxiety (indicated by GAD-7 score ≥ 5), and 11.4% (n = 11/96) reported moderate to severe depressive symptoms (PHQ-9 score ≥ 10). A minority (n = 3/96, 3.1%) reported symptoms consistent with probable PTSD (PC-PTSD-5 score 4–5) [Figure 1].

The majority of patients (n = 73/96, 76%) who participated in the study felt that the COVID-19 pandemic had little or no effect on their mental health, although 84.4% (n = 81/96) were concerned to some extent that they might be exposed to the virus [Table 2]. Only a minority (n = 6/96, 6.3%) felt that they wanted more mental health support during COVID-19, and the vast majority (n = 91/96, 94.8%) did not receive or want any mental health support [Table 2]. One in two participants (n = 48/96, 50%) felt that COVID-19 did not have a negative impact on their cancer treatment [Table 2]. Almost all patients (n = 95/96, 99%) considered being able to have cancer treatment to be more important than the threat of COVID-19 infection [Table 2]. The main concerns around cancer treatment focused on the risk of relapse or progression (n = 20/96, 20.8%) and the uncertainty around when treatment or tests would restart (n = 15/96, 15.6%) [Table 2].

**Figure 2:**
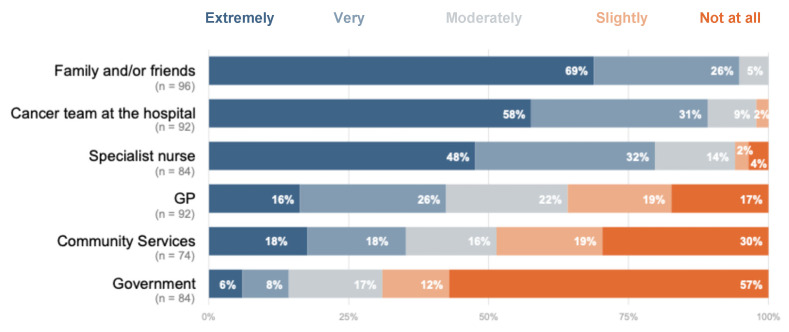
Levels of support participants reported they had received during the COVID-19 pandemic (n = 96). See Supplementary Table S2 for further details.

Participants reported the use of various personal coping strategies to manage their emotional well-being during the survey period. The most commonly endorsed ones were focusing on positives (n = 39/96, 40.6%), using humour (n = 27/96, 28.1%), changing physical activity (n = 22/96, 22.9%), and avoiding thinking about it (n = 20/96, 20.8%) [Table 2]. Friends and family were an important source of support for most patients, with 94.8% (n = 91/96) reporting these people being extremely or very supportive [Figure 2]. Healthcare providers, including the cancer team (n = 82/92, 89.1%) and specialist nurses (n = 67/84, 79.8%), provided high levels of support, but the perception of support from community services (n = 26/74, 35.1%) and the government (n = 12/84, 14.3%) was lacking [Figure 2].

### 3.3. Factors Associated with Anxiety, Depressive Symptoms, Trauma-Related Symptoms, and Poor Well-Being

In the multivariate analysis, male participants were at a lower risk of poor well-being compared to female participants (OR 0.27, CI 0.07–0.996; *p* = 0.049) [Table 3 and Supplementary Table S1]. Participants who were concerned that COVID-19 would have a negative impact on their cancer treatment (OR 4.59, 95% CI 1.03–20.56; *p* = 0.046) or those who reported that the COVID-19 pandemic had affected their mental health more than moderately (OR 3.90, 95% CI 1.38–11.03; *p* = 0.010) had a higher probability of reporting poor well-being (WHO-5 < 50) [Table 3 and Supplementary Table S1].

Participants who were concerned that the pandemic would affect their mental health were more likely to be at risk of anxiety (GAD-7 score ≥ 5) (OR 3.82, 95% CI 1.96–7.44; *p* < 0.001) [Table 3 and Supplementary Table S1]. Concerns regarding interruptions in cancer care, including cancelled surveillance scans and backlogs, were also associated with higher probability of anxiety (OR 9.22, 95% CI 1.09–77.85; *p* = 0.041) [Table 3 and Supplementary Table S1]. No factors were found to have a statistically significant effect on the risk of depression (PHQ-9 score ≥ 10) or PTSD [Supplementary Table S1].

### 3.4. Longitudinal Comparison in the Mental Health Status of Cohort 2 Participants

Fifty-seven participants (65.5%) consented to complete the same questionnaire at time point 2, 6 months after the initial survey. There was an increase in the number of participants who had tested positive for COVID-19 infection (n = 2/57, 3.5% vs. n = 14/57, 24.6%; *p* = 0.003). In a longitudinal pairwise comparison, there was no difference in the mental health status of participants within the 6 months period (*p* > 0.05): poor well-being (n = 20/57, 35.1% vs. n = 15/57, 26.3%), anxiety (n = 17/57, 29.8% vs. n = 14/57, 24.6%), and depressive symptoms (n = 9/57, 15.8% vs. n = 7/57, 12.3%) [Figure 1E].

### 3.5. Comparison in the Mental Health Status of Cohort 1 Vs. Cohort 2 Participants

We compared the previously reported outcome measures of the participants included in Cohort 1 of PICO-SM [11], recruited between 7 and 28 April 2021, with the current Cohort 2 to investigate if there were any potential changes in the impact of COVID-19 pandemic on patients over time [Supplementary Table S2]. In terms of demographics, in Cohort 2, there were more male participants (63.5% vs. 56.5%, *p* < 0.001), and more participants reported a previous diagnosis of mental health illness (33.3% vs. 21.8%, *p* = 0.018) [Supplementary Table S2]. Taking into account the minor differences in demographic features between the cohorts, there were no statistically significant differences in the proportion of patients with poor well-being, risk of depression, anxiety, or probable PTSD [Supplementary Table S3]. Despite this, fewer participants in Cohort 2 endorsed the use of coping mechanisms (77.7% vs. 82.4%, *p* = 0.005) [Supplementary Table S2]. The majority of participants persistently expressed more concerns about their cancer treatment than the risk of COVID-19 infection, including uncertainties regarding when treatment or tests would restart [Supplementary Table S2].

## 4. Discussion

PICO–SM was a large prospective longitudinal study assessing the impact of the COVID-19 pandemic, at two time points (2021–2022), on the mental health burden in patients with colorectal cancer. Following the initial results of our institutional audit [3], this subsequent PICO-SM two-part study was conducted to identify how psychological distress and well-being were affected throughout the course of the pandemic.

In our prospective survey series, levels of poor well-being, anxiety, and depressive symptoms remained similar throughout the study period for participants in both Cohorts 1 and 2 [11]. Although there is a paucity of historical data on the prevalence of psychological distress in our local population, it appears that the levels of anxiety and depression in both our PICO-SM cohorts were similar to those estimated in other studies prior to the COVID-19 pandemic [6]. Additionally, the reported levels of trauma-related symptoms remained remarkably low in our series, with no signs of fluctuation throughout waves of the pandemic, in contrast to other studies reporting rates of PTSD up to 30% [9,17].

In preliminary studies conducted at our institution in the first two years of the pandemic [3,4,11], we identified several common themes associated with an increased risk of psychological distress, as well as collected data on which of these were most important to patients with colorectal cancer. Participants who expressed concerns that the pandemic would affect their mental health had an increased risk of anxiety [3,11] and, in the current study, depressive symptoms [11] or well-being [11]. Those who reported a need for more support were at a higher risk of experiencing anxiety [3,11]. Despite the individual stressors identified in our study, the continuation of service delivery was the most important factor for patients undergoing oncological management. Although the COVID-19 pandemic is now no longer considered a public health emergency of international concern (it is now an established and ongoing health issue; WHO, May 2023), the invaluable lessons we learned should prepare public health responses to similar conditions in the future. Identifying the population in need, especially those with pre-existing mental health conditions or those with an increased risk of psychological distress according to psychometric tools, could facilitate referral to the appropriate support services.

During the COVID-19 pandemic, different coping mechanisms were endorsed by the participants, and the support of friends, family, and the healthcare team was deemed very important for them. Nevertheless, in both cohorts of PICO-SM, the participants did not appear to be satisfied with the support received from the government during the COVID-19 pandemic period. We cannot ascertain whether this was due to the specific geographical distribution in which the study was carried out or a wider national or global perception. Although similar results are not available for patients with cancer, similar sentiments regarding the support received from the respective governments were also reported by oncology professionals enrolled in the European Society of Medical Oncology (ESMO) Resilience Task Force global oncology survey series [18].

To future-proof healthcare systems and enhance patient experience, a comprehensive strategy is essential. The American Society of Clinical Oncology (ASCO) and the ESMO guidelines both recommend the use of PHQ-9 and GAD-7 for the screening and assessment of depression and anxiety in patients with cancer [19,20]. Although population-wide screening for common mental health conditions offers potential benefits in terms of early detection and prevention, it is important to consider the potential for over-diagnosis, over-treatment, and demographic variations in prevalence in the design and implementation of screening programmes [21]. A greater emphasis on preventative care through public health initiatives and patient education can also reduce the burden of chronic diseases and system pressures. Engaging patients as active participants in their care through transparent communication and shared decision making is vital, significantly enhancing satisfaction and outcomes. By focusing on these key areas, healthcare systems can become more adaptive and effective in meeting future demands while ensuring an improved patient experience.

### Limitations

Our study was limited by the inclusion of patients with colorectal cancer from a tertiary cancer centre, and our results may not reflect the experience of cancer units that lack specialised support such as psycho-oncology services. Our centre’s setup for comprehensive cancer care may in part be the reason for the low rates of psychological burden. The response rate to this survey (39%) was lower than expected, which is likely a result of the survey fatigue observed during the pandemic [22]. Despite the relatively small size of Cohort 2, in total, the lived experiences of more than n = 310 patients were prospectively studied throughout PICO-SM. Finally, self-reported outcome measures were used in the study design, which may need further objective validation in future studies.

## 5. Conclusions

The COVID-19 pandemic undeniably posed an immense challenge for both patients with cancer and healthcare systems and providers globally. The two cohorts of PICO-SM revealed that, whilst there was a subgroup of patients with colorectal cancer who were at a higher risk of poor well-being, anxiety, and/or depression, the primary concerns consistently reported by the majority of the participants were pertaining to issues critical to their respective cancer management, especially with regard to tests and treatments. This highlights that, in future crises, robust national and local contingency plans should be put in place to ensure that cancer care for patients is resilient. Further studies are essential to leverage our current understanding on best-practice improvements that can better support our patients.

## Figures and Tables

**Table 1:** Baseline demographics of participants in PICO-SM Cohort 2 (n = 96).

	Number, n (%)
**Gender**	
Male	61 (63.5)
Female	34 (35.4)
Other	1 (0.1%)
**Mean Age (years)**	64.0 ± 10.7
**Ethnicity**	
White/White British	89 (92.7)
Other	7 (7.3)
**Marital Status**	
Single/Divorced/Separated/Widowed	22 (22.9)
In a relationship/Married/In civil partnership	73 (76.0)
*Did not answer*	1 (1.0)
**Have children**	
Yes	79 (82.3)
No	16 (16.7)
*Did not answer*	1 (1.0)
**Live alone**	
Yes	17 (17.7)
No	78 (81.3)
*Did not answer*	1 (1.0)
**Previous/Underlying diagnosis of mental health condition ^a^**	
Anxiety	19 (19.8)
Depression	17 (17.7)
Panic attacks	6 (6.3)
Post-traumatic stress disorder	4 (4.2)
Other	4 (4.2)
**None of the above**	64 (66.7)
**Conditions and/or comorbidities which may increase personal risk of being ill with COVID-19**	
Yes	30 (31.3)
No	34 (35.4)
*Prefer not to say*	32 (33.3)
**Have had testing for COVID-19**	
**Yes**	91 (94.8)
Tested positive	13/91 (14.3)
**No**	4 (4.2)
*Did not answer*	1 (1.0)
**Have had COVID-19 requiring hospitalisation**	
Yes	3 (3.1)
No	89 (92.7)
*Did not answer*	4 (4.2)

^a^ More than one response permitted.

**Table 2:** The impact of the COVID-19 pandemic on patients with colorectal cancer and coping mechanisms—Cohort 2 of PICO-SM (n = 96).

	Number, n (%)
**Concerned that COVID-19 had/will have a negative impact on their cancer treatment**	
Yes	34 (35.4)
No	48 (50.0)
Do not know	14 (14.6)
**More concerned about COVID-19 rather than their cancer**	
Yes	1 (1.0)
No	95 (99.0)
**Key concerns about cancer treatment and care during COVID-19 pandemic ^a^**	
Concerned cancer will come back or progress while waiting for treatment	20 (20.8)
Where to get help with dealing with side effects	5 (5.2)
Uncertainty around when treatment or tests will restart	15 (15.6)
Lack of contact with clinical team	5 (5.2)
Who to contact if felt cancer has come back or spread	8 (8.3)
Surveillance scans cancelled and concern over backlog	6 (6.3)
**Felt COVID-19 pandemic has affected mental health**	
Extremely	3 (3.1)
Very much	5 (5.2)
Moderately	15 (15.6)
Slightly	29 (30.2)
Not at all	44 (45.8)
**Wanted more support for mental health during COVID-19**	
Yes	6 (6.3)
No	87 (90.6)
*Prefer not to say*	3 (3.1)
**Have received support from primary cancer hospital for mental health during COVID-19**	
Yes	3 (3.1)
No and Did not need support	91 (94.8)
*Prefer not to say*	2 (2.1)
**Personal coping strategies**	
**Yes ^a^**	
Focusing on positives	115 (53.2)
Using humour	84 (38.9)
Change in physical activity (e.g., exercise)	73 (33.8)
Avoiding thinking about it	66 (30.6)
Planning time	50 (23.1)
Distracting self	43 (19.9)
Changes in diet (e.g., types of food, amount)	28 (13.0)
Using religious or spiritual practice(s)	26 (12.0)
Talking to medical professions	24 (11.1)
Using meditation, mindfulness or other relaxation techniques	21 (9.7)
Changing substance intake (e.g., smoking, alcohol, other drugs)	11 (5.1)
Other	2 (0.9)
**None of the above**	38 (17.6)

^a^ More than one response permitted.

**Table 3:** Univariate and multivariate analyses of factors associated with an increased risk of poor well-being (WHO-5) and anxiety (GAD-7) (n = 96).

Variable	Univariate Analysis			Multivariate Analysis		
	Odds Ratio	95% CI	*p* Value	Odds Ratio	95% CI	*p* Value
**Factors associated with poor well-being (WHO < 50)**						
**Gender (M/F)**	0.33	0.13–0.79	0.012	0.27	0.07–0.996	**0.049**
Concern might get COVID-19	1.57	0.995–2.47	0.052	0.38	0.14–1.01	0.053
**Concerned that COVID-19 had/will have a negative impact on their cancer treatment**	3.36	1.30–8.71	0.011	4.59	1.03–20.56	**0.046**
**Affect on mental health**	2.74	1.65–4.56	<0.001	3.90	1.38–11.03	**0.010**
Mental health affects care	4.30	1.15–16.06	0.037	0.10	0.01–1.75	0.113
Want more support	13.13	1.46–118.21	0.011	36.55	0.40–3350.23	0.119
Change in physical activity (e.g., exercise)	0.25	0.07–0.90	0.038	0.22	0.04–1.16	0.074
**Factors associated with increased anxiety (** **GAD ≥ 7** **)**						
**Surveillance scans cancelled and concern over backlog**	6.09	1.04–35.56	0.046	9.22	1.09–77.85	**0.041**
**Affect on mental health**	3.30	1.89–5.77	<0.001	3.82	1.96–7.44	**<0.001**

## Data Availability

All data presented in this study are contained within the article and Supplementary Materials. Any further data, which might have been omitted due to privacy or ethical restrictions, may be made available upon reasonable request.

## Supplementary Materials

The following supporting information can be downloaded at https://www.mdpi.com/article/10.3390/curroncol31120582/s1: Table S1: Univariate and multivariate analyses of factors associated with poor well-being (WHO-5 < 50), anxiety (GAD-7 ≥ 5), and depression (PHQ-9 ≥ 10); Table S2: Comparison of all the characteristics of Cohorts 1 and 2 of PICO-SM; Table S3: Comparison of all the key outcome variables of Cohorts 1 and 2 of PICO-SM.
