# Supplementary material for: Longitudinal Follow-Up of the Psychological Well-Being of Patients with Colorectal Cancer: Final Analysis of PICO-SM
Source: Curr Oncol. 2024 Dec 11;31(12):7903–13. doi: 10.3390/curroncol31120582 (PMC11674373; doi:10.3390/curroncol31120582)
Supplement: Supplementary file 1 [file curroncol-31-00582-s001.zip › curroncol-3290345-supplementary.pdf]

SUPPLEMENTARY DATA

Supplementary Table S1. Univariate and multivariate analyses of factors associated with a poor well-being (WHO-5 < 50), anxiety (GAD-7 ≥ 5), and depression (PHQ-9 ≥ 10).

Variable	Univariate Analysis			Multivariate Analysis		
	**Odds Ratio**	**95% CI**	**p-Value**	**Odds Ratio**	**95% CI**	**p-Value**
**Factors associated with a poor well-being (WHO- 5 < 50)**						
**Gender (M/F)**	3.07	1.26-7.47	0.012	0.27	0.07-0.996	**0.049**
Past history: anxiety	2.73	0.98-7.62	0.051			
Past history: depression	3.70	1.25-10.94	0.014			
Past history: panic attacks	11.67	1.30-104.65	0.015			
Past history: post-traumatic stress disorder			0.011			
Past history: none	0.27	0.11-0.67	0.004			
Concerned they might get COVID-19	1.57	0.995-2.47	0.052	0.38	0.14-1.01	0.053
**Concerned that COVID-19 had/will have a negative impact on their cancer treatment**	3.36	1.30-8.71	0.011	4.59	1.03-20.56	**0.046**
**Effect on mental health**	2.74	1.65-4.56	<0.001	3.90	1.38-11.03	**0.010**
Mental health affects care	4.30	1.15-16.06	0.037	0.10	0.01-1.75	0.113
Want more support	13.13	1.46-118.21	0.011	36.55	0.40-3350.23	0.119
Change in physical activity (e.g., exercise)	0.25	0.07-0.90	0.038	0.22	0.04-1.16	0.074
**Factors associated with increased anxiety (GAD -7 ≥5)**						
Gender (M/F)	2.28	0.91-5.75	0.076			
Age (years)	0.941	0.898-0.986	0.011			
Past history: nervous/anxious	4.17	1.45-11.98	0.006			
Past history: depression	4.10	1.38-12.24	0.008			
Past history: panic attacks	6.18	1.06-36.08	0.044			
Past history: post-traumatic stress disorder			0.005			
Past history: none of the above	0.24	0.09-0.61	0.002			
Have had COVID-19 requiring hospitalisation			0.018			
Concerned they might get COVID-19	1.84	1.11-3.04	0.017			
Concerned that COVID-19 had/will have a negative impact on their cancer treatment			0.019			
Who to contact if felt cancer has come back or spread	5.24	1.15-23.79	0.033			
**Surveillance scans cancelled and concern over backlog**	6.09	1.04-35.56	0.046	9.22	1.09-77.85	**0.041**
**Effect on mental health**	3.30	1.89-5.77	<0.001	3.82	1.96-7.44	**<0.001**

Mental health affects care	11.17	2.66-46.88	<0.001			
Want more support			<0.001			
Coping: distracting self	3.10	0.82-11.75	0.085			
Coping: using meditation, mindfulness or other relaxation techniques	3.10	0.82-11.75	0.085			
Coping: changing substance intake (e.g., smoking, alcohol, other drugs)	4.06	0.84-19.57	0.083			
Coping: none of the above	0.40	0.14-1.20	0.095			
**Factors associated with increased depression (PHQ-9 ≥ 10)**						
Gender (M/F)	5.95	1.46-24.25	0.015			
Age	0.937	0.879-0.998	0.043			
Past history: nervous/anxious	6.55	1.74-24.68	0.002			
Effect on mental health	2.37	1.58-3.54	<0.001			
Past history: depression	5.07	1.34-19.26	0.010			
Past history: post-traumatic stress disorder	31.5	2.93-339.17	0.004			
Past history: none of the above	0.08	0.02-0.41	<0.001			
Self-reported perception of current status of cancer: progressive disease/stable disease	8.17	1.19-56.00	0.044			
Self-reported perception of current status of cancer: unknown/stable disease	5.44	1.03-28.86	0.030			
Have had COVID-19 requiring hospitalisation	17.78	1.46-216.02	0.037			
Concerned they might get COVID-19	3.20	1.43-7.15	0.005			
Felt the COVID-19 pandemic has affected mental health	4.51	2.11-9.66	<0.001			
Mental health affect care	13.00	2.92-57.80	<0.001			
Want more support	32.80	7.80-224.30	<0.001			
Coping: change in physical activity			0.064			
Coping: distracting self	4.18	0.90-19.41	0.086			
Coping: changes in diet	3.47	0.88-13.71	0.083			
Coping: using meditation, mindfulness or other relaxation techniques	4.18	0.90-19.41	0.086			

Supplementary Table S2. Comparison of all the characteristics of Cohorts 1 and 2 of the PICO-SM study.

	Number, *n* (%)<br>Or<br>Mean ± SD		Univariate analysis	
	**Cohort 1**	**Cohort 2 (ref)**	**OR with 95 CI**	**Method**
**Gender**<br>Male<br>Female<br>Other<br>*Prefer not to say*	 122 (56.5)<br>91 (42.1)<br>1 (0.5)<br>2 (0.9)	 61 (63.5)<br>34 (35.4)<br><br>1 (0.1)	Ref.<br>**0.75**<br>**1.82–4.02**<br>**P<0.001**	Chi-square test<br>(male vs. female)
**Mean age (years)**	65.3±10.2	64.0±10.7	1.01<br>0.99–1.04<br>p=0.337	Binary logistic regression
**Ethnicity**<br>White/White British<br>Asian/Asian British (Indian, Pakistani, Bangladeshi)<br>Asian/Asian British (Chinese)<br>Black/Black British<br>Mixed<br>Other<br>*Prefer not to say*	 198 (91.7)<br>5 (2.3)<br><br>1 (0.5)<br>3 (1.4)<br>2 (0.9)<br>5 (2.3)<br>2 (0.9)	 89 (92.7)<br>1 (1.0)<br><br>2 (2.1)<br>1 (1.0)<br>1 (1.0)<br>1 (1.0)<br>1 (1.0)	1.01<br>0.44–2.33<br>p=0.966	Chi-square test<br>(white vs. all/others)
**Marital status**<br>Single/ divorced /separated/ widowed<br>In a relationship/married/ in a civil partnership<br>*NA*	 70 (32.4)<br>144 (66.7)<br>2 (0.9)	 22 (22.9)<br>73 (76.0)<br>1 (1.0)	0.62<br>0.35–1.07<br>p=0.092<br>Ref	Chi-square test<br>(single/ divorced /separated/ widowed **vs.**<br>in a relationship/ married/in a civil partnership)
**Has children**<br>Yes<br>No<br>*NA*	 168 (77.8)<br>44 (20.4)<br>4 (1.9)	 79 (82.3)<br>16 (16.7)<br>1 (1.0)	0.77<br>0.40-1.43<br>p=0.425	Chi-square test<br>(yes/no)
**Lives alone**<br>Yes<br>No<br>*NA*	 51 (23.6)<br>162 (75.0)<br>3 (1.4)	 17 (17.7)<br>78 (81.3)<br>1 (1.0)	1.44<br>0.80–2.72<br>p=0.239	Chi-square test<br>(yes/no)

**Previous/underlying diagnosis of mental health condition**[a]				Chi-square test or Fisher's exact test
Anxiety	25 (11.6)	19 (19.8)	0.54<br>0.28–1.04<br>p=0.063	
**Depression**	18 (8.3)	17 (17.7)	**0.43**<br>**0.21–0.89**<br>**p=0.021**	
Panic attacks	9 (4.2)	6 (6.3)	0.67<br>0.23–2.05<br>p=0.457	
Anorexia	0(0.0)	0 (0.0)		
Psychosis	0(0.0)	1 (1.0)		
Bulimia	0(0.0)	0 (0.0)		
Social phobia	1 (0.5)	1 (1.0)		
Attention deficit disorder	1 (0.5)	0(0.0)		
Obsessive compulsive disorder	0(0.0)	1(1.0)		
Autism	0(0.0)	0(0.0)		
Post-traumatic stress disorder	2 (0.9)	4 (4.2)	0.22<br>0.03–1.15<br>p=0.084[F]	
Alcohol/drugs	1 (0.5)	1 (1.0)		
Bipolar disorder	1 (0.5)	0 (0.0)		
Personality disorder	1 (0.5)	0 (0.0)		
Other	1 (0.5)	1 (1.0)		
**None of the above**	169 (78.2)	64 (66.7)	**1.91**<br>**1.13–3.24**<br>**p=0.018**	
**Self-reported perception of current status of cancer**[a]				Chi-square test
Stable disease (shrinking/responding well to treatment, under control or stable, has finished treatment and has routine monitoring scans/check-ups, has had curative treatment and no active cancer)	126 (58.3)	51 (53.1)	Ref.	
Progressive disease (progressing/getting worse)	33 (15.3)	12 (12.5)	1.11<br>0.54–2.40<br>p=0.776	
Unknown (Recently diagnosed and waiting for treatment to start, undergoing investigations at this stage, Does not know or is not certain, other, *prefer not to say)*	57 (26.4)	33 (34.4)	0.59<br>0.34–1.03<br>p=0.063	
**Conditions and/or comorbidities which may increase personal risk of being ill with COVID-19**				Chi-square test (yes/no)

Yes<br>*No*<br>*Prefer not to say*	67 (31.0)<br>125 (57.9)<br>24 (11.1)	30 (31.3)<br>34 (35.4)<br>32 (33.3)	**0.29**<br>**0.18–0.48**<br>**p<0.001**	
**Would like to have testing for COVID-19, *n* = 60**<br>Yes<br>No	 <br>62 (39.2)<br>96 (60.8)	 <br>22 (36.7)<br>38 (63.3)	 1.12<br>0.61–2.08<br>p=0.727	Chi-square test
**Have had testing for COVID-19**<br>Yes<br>Tested positive<br>No<br>*Did not answer*	 171 (79.2)<br>9/171 (5.3)<br>43 (19.9)<br>2 (0.9)	 91 (94.8)<br>13/91 (14.3)<br>4 (4.2)<br>1 (1.0)	 **0.17**<br>**0.05 – 0.45**<br>**p=0.001**	Fisher's exact test (yes/no)
**Have had COVID-19 requiring hospitalisation**<br>Yes<br>No<br>*Did not answer*	 <br>3 (1.4)<br>203 (94.0)<br>10 (4.6)	 <br>3 (3.1)<br>89 (92.7)<br>4 (4.2)	 <br>0.44<br>0.08–2.41<br>p=0.318	Fisher's exact test (yes/no)
**Concerned they might get COVID-19**<br>Extremely<br>Very much<br>Moderately<br>Slightly<br>Not at all<br>*Did not answer*	12 (5.6)<br>18 (8.3)<br>48 (22.2)<br>78 (36.1)<br>54 (25.0)<br>6 (2.8)	2 (2.1)<br>14 (14.6)<br>38 (39.6)<br>27 (28.1)<br>15 (15.6)<br>0 (0.0)	**0.79**<br>**0.63–0.99**<br>**p=0.038**	Binary logistic regression
**Concerned that COVID-19 had/will have a negative impact on their cancer treatment (Q10)**<br>Yes<br>No<br>Don't know<br>*Did not answer*	 <br><br>58 (26.9)<br>128 (59.3)<br>27 (12.5)<br>3 (1.4)	 <br><br>34 (35.4)<br>48 (50.0)<br>14 (14.6)<br>0 (0.0)	 <br><br>0.64<br>0.38–1.10<br>p=0.107	Chi-square test (Yes/No)
**More concerned about COVID-19 rather than their cancer**<br>Yes<br>No<br>*Did not answer*	 <br>14 (6.5)<br>193 (89.4)<br>9 (4.2)	 <br>1 (1.0)<br>95 (99.0)<br>0 (0.0)	 <br>6.89<br>1.35–125.81<br>p=0.064	Fisher's exact test
**Key concerns about cancer treatment and care during COVID-19 pandemic**[a]<br>Concerned cancer will come back or progress while waiting for treatment<br><br>Where to get help with dealing with side effects<br><br>**Uncertainty around when treatment or tests will restart**<br><br>Lack of contact with clinical team	 <br><br>37 (17.1)<br><br><br><br>16 (7.4)<br><br><br><br>14 (6.5)	 <br><br>20 (20.8)<br><br><br><br>5 (5.2)<br><br><br><br>15 (15.6)	 <br><br>0.85<br>0.47–1.59<br>p=0.604<br><br>1.57<br>0.59–4.91<br>p=0.396<br><br>**0.40**<br>**0.18–0.87**<br>**p=0.021**	Chi-square test

Who to contact if felt cancer has come back or spread	12 (5.6)	5 (5.2)	1.14<br>0.41 – 3.68<br>p=0.808	
Surveillance scans cancelled and concern over backlog	10 (4.6)	8 (8.3)	0.57<br>0.22–1.55<br>p=0.256	
	9 (4.2)	6 (6.3)	0.70<br>0.24–2.14<br>p=0.509	
**Felt COVID-19 pandemic has affected mental health**				
Extremely	3 (1.4)	3 (3.1)	0.95 (0.75–1.21)<br>p=0.691	Binary logistic regression
Very much	11 (5.1)	5 (5.2)		
Moderately	39 (18.1)	15 (15.6)		
Slightly	53 (24.5)	29 (30.2)		
Not at all	102 (47.2)	44 (45.8)		
Did not answer	8 (3.7)			
**Mental health has affected experience of cancer care (q15)**				Chi-square test<br>(yes/no)
Yes	24 (11.1)	11 (11.5)	0.99	
No	183 (84.7)	83 (86.5)	0.47–2.19	
Prefer not to say	9 (4.2)	2 (2.1)	p=0.978	
**Have received support from primary cancer hospital for mental health during COVID-19**				Fisher's Exact test<br>(Yes/No& Did not need support)
Yes	10 (4.6)	3 (3.1)	1.52	
No and did not need support	199 (92.2)	91 (94.8)	0.45–6.92	
Prefer not to say	7 (3.3)	2 (2.1)	p=0.529	
**Wanted more support for mental health during COVID-19**				Fisher's Exact test<br>(Yes/No)
Yes	15 (6.9)	6 (6.3)	1.13	
No	192 (88.9)	87 (90.6)	0.44–3.27	
Prefer not to say	9 (4.2)	3 (3.1)	p=0.803	
**Personal coping strategies**				
**Yes**[a]				
**Focusing on positives**	115 (53.2)	39 (40.6)	**1.68**<br>**1.04–2.75**<br>**p=0.037**	
Using humour	84 (38.9)	27 (28.1)	1.64<br>0.98–2.79<br>p=0.064	Chi-square test<br>Or<br>Fisher's exact test
**Change in physical activity (e.g., exercise)**	73 (33.8)	22 (22.9)	**1.73**<br>**1.01–3.06**<br>**p=0.050**	
Avoiding thinking about it	66 (30.6)	20 (20.8)	1.68<br>0.96–3.04	

			p=0.074	
**Planning time**	50 (23.1)	13 (13.5)	**1.93**<br>**1.02–3.90**<br>**p=0.050**	
**Distracting self**	43 (19.9)	10 (10.4)	**2.15**<br>**1.07–4.72**<br>**p=0.041**	
Changes in diet (e.g., types of food, amount)	28 (13.0)	16 (16.7)	0.75<br>0.39–1.49<br>p=0.396	
Using religious or spiritual practice(s)	26 (12.0)	6 (6.3)	2.06<br>0.87–5.70<br>p=0.124	
Talking to medical professions	24 (11.1)	7 (7.3)	1.60<br>0.70–4.14<br>p=0.296	
Using meditation, mindfulness or other relaxation techniques	21 (9.7)	10 (10.4)	0.93<br>0.43–2.14<br>p=0.860	
Changing substance intake (e.g., smoking, alcohol, other drugs)	11 (5.1)	7 (7.3)	0.69<br>0.26–1.92<br>p=0.450	
Other	2 (0.9)	0 (0.0)		
**None of the above**	**38 (17.6)**	**31 (32.3)**	**0.45**<br>**0.26–0.78**<br>**p=0.005**	
**Support: cancer team**<br>Extremely<br>Very much<br>Moderately<br>Slightly<br>Not at all<br>*Did not answer*	 112 (51.9)<br>60 (27.8)<br>19 (8.8)<br>8 (3.7)<br>5 (2.3)<br>12 (5.6)	 53 (55.2)<br>29 (30.2)<br>8 (8.3)<br>2 (2.1)<br>0 (0.0)<br>4 (4.2)	0.83<br>0.61–1.10<br>p=0.214	Binary logistic regression
**Support: specialist nurse**<br>Extremely<br>Very much<br>Moderately<br>Slightly<br>Not at all<br>*Did not answer*	 78 (36.1)<br>44 (20.4)<br>19 (8.8)<br>8 (3.7)<br>26 (12.0)<br>41 (19.0)	 40 (41.7)<br>27 (28.1)<br>12 (12.5)<br>2 (2.1)<br>3 (3.1)<br>12 (12.5)	**0.79**<br>**0.63–0.97**<br>**p=0.032**	

**Support: my GP**				
Extremely	44 (20.4)	15 (15.6)		
Very much	29 (13.4)	24 (25.0)	0.93	
Moderately	41 (19.0)	20 (20.8)	0.79–1.10	
Slightly	20 (9.3)	17 (17.7)	p=0.414	
Not at all	58 (26.9)	16 (16.7)		
Did not answer	24 (11.1)	4 (4.2)		
**Support: community services**				
Extremely	41 (19.0)	13 (13.5)		
Very much	25 (11.6)	13 (13.5)	1.05	
Moderately	30 (13.9)	12 (12.5)	0.88–1.25	
Slightly	23 (10.6)	14 (14.6)	p=0.616	
Not at all	55 (25.5)	22 (22.9)		
Did not answer	42 (19.5)	22 (22.9)		
**Support: government**				
Extremely	14 (6.5)	5 (5.2)		
Very much	30 (13.9)	7 (7.3)	**1.48**	
Moderately	53 (24.5)	14 (14.6)	**1.19–1.85**	
Slightly	36 (16.7)	10 (10.4)	**p=0.001**	
Not at all	54 (25.0)	48 (50.0)		
Did not answer	29 (13.5)	12 (12.5)		
**Support: friends/family**				
Extremely	153 (70.8)	66 (68.8)		
Very much	40 (18.5)	25 (26.0)	0.97	
Moderately	7 (3.2)	5 (5.2)	0.68–1.34	
Slightly	3 (1.4)	0 (0.0)	p=0.850	
Not at all	4 (1.9)	0 (0.0)		
Did not answer	9 (4.2)			

[a] more than one option to the question possible

Supplementary Table S3. Comparison of all the key outcome variables of Cohorts 1 and 2 of the PICO-SM study.

	**Number, *n* (%) Or Mean ± SD**		**Univariate analysis**	**Method**
	**Cohort 1**	**Cohort 2**	**OR with 95 CI**	
**Anxiety GAD-7 score (≥5)**				
Yes	48 (22.2)	26 (27.1)	0.83<br>048–1.45<br>p=0.521	Chi-square test (yes/no)
No	155 (71.8)	70 (72.9)		
Missing	13 (6.0)	0 (0)		
**Depression PHQ-9 score (≥10)**				
Yes	31 (14.4)	11 (11.5)	1.39<br>0.66 2.89<br>p=0.394	Chi-square test (yes/no)
No	173 (80.1)	85 (88.5)		
Missing	12 (5.6)	0 (0)		
**Wellbeing WHO-5 (<50)**				
Yes	66 (30.6)	32 (33.3)	0.90<br>0.54–1.51<br>p=0.681	Chi-square test (yes/no)
No	147 (68.1)	64 (66.7)		
Missing	3 (1.4)	0 (0)		
**PTSD score (≥4)**				
Yes	5 (2.3)	3 (3.1)	0.84<br>0.20–3.58<br>p=0.999	Fisher's exact test (yes/no)
No	185 (85.6)	93 (96.9)		
Missing	26 (12.0)	0 (0)		
